# Differential effects of prenatal psychological distress and positive mental health on offspring socioemotional development from infancy to adolescence: a meta-analysis

**DOI:** 10.3389/fped.2023.1221232

**Published:** 2023-09-14

**Authors:** Desiree Y. Phua, Chermaine S. M. Chew, Yang Lik Tan, Benjamin J. K. Ng, Florence K. L. Lee, Megan M. Y. Tham

**Affiliations:** ^1^ Singapore Institute for Clinical Sciences (SICS), Agency for Science, Technology and Research (A*STAR), Singapore, Singapore; ^2^ School of Management and Communication, Republic Polytechnic, Singapore, Singapore

**Keywords:** prenatal mental health, positive mental health, socioemotional development, perinatal influence, depression, early life, anxiety

## Abstract

The impact of prenatal maternal mental health on offspring socioemotional development is substantial and enduring. Existing literature primarily focuses on the effects of psychological distress during pregnancy, emphasizing adverse child outcomes. Recent studies, however, highlight the unique impact of positive maternal mental health on child outcomes. To elucidate the differential associations of maternal psychological distress and positive mental health during pregnancy with child outcomes, we conducted a systematic literature search and random-effects meta-analyses on studies investigating the associations of prenatal maternal mental health with child socioemotional development. Our analyses, comprising 74 studies with 321,966 mother-child dyads across 21 countries, revealed significant associations of prenatal psychological distress with both adverse and positive child socioemotional outcomes. Notably, the effect sizes for the association of psychological distress with positive child outcomes were smaller compared to adverse outcomes. Positive prenatal mental health, on the other hand, was significantly associated with positive socioemotional outcomes but not adverse outcomes. This meta-analysis highlights the independence of negative and positive prenatal mental health constructs and their distinct relationships with child socioemotional development. The findings underscore the importance of considering the positive spectrum of maternal mental health and developmental outcomes to enhance our understanding of prenatal influences on child development.

**Systematic Review Registration:** https://www.crd.york.ac.uk/prospero/display_record.php?RecordID=335227, identifier CRD42022335227.

## Introduction

The impact of maternal mental health on child outcomes is of global significance (1, 2). Notably, the annual economic burden of inadequate perinatal mental health in the United Kingdom has been approximated at £8.1 billion (3). Depressive symptoms experienced by mothers during pregnancy are strongly associated with the risk of depression in the offspring (4, 5). Beyond depression, fetal development is intricately shaped by a diverse spectrum of prenatal mental health factors. Furthermore, it is worth noting that the ramifications of prenatal mental health on offspring persist even when accounting for postpartum maternal mental well-being (6, 7).

Nonetheless, it is crucial to recognize that mental health encompasses more than the mere absence of mental disorders. The World Health Organization (8) defines health as the state of “complete physical, mental, and social well-being and not merely the absence of disease or infirmity” (p. 1). Negative and positive mental health, far from being opposing extremes along a single continuum, are discrete constructs characterized by distinct biological correlates, antecedents, and outcomes (9–11). Children grappling with symptoms of mental illness might concurrently exhibit positive mental health marked by favorable psychological attributes (12). Similarly, the absence of mental illness may not necessarily signify a high level of positive mental health or overall mental well-being (13). As such, it becomes imperative to explore both the domains of negative and positive mental health to understand holistically how maternal mental health influences offspring socioemotional development.

Furthermore, a growing body of evidence reveals that the determinants of negative and positive dimensions of child development diverge distinctly. Parental mental health predicted mental ill-health but not well-being, while school connectedness and friendships predicted well-being in children but not poor mental health (14). A recent twin study echoed this divergence, revealing that factors safeguarding individuals from mental illnesses differ substantially from those fostering well-being (15). Adverse early life circumstances may influence negative child outcomes, such as emotional or behavioral difficulties, without extending the same impact to positive well-being (16). Despite emerging publications on this subject [e.g., (17–19)], the potential influence of positive states within maternal mental health remains predominantly overlooked. Therefore, our focus lies in investigating whether maternal psychological distress and positive mental health during pregnancy exert dissimilar effects on the distinct domains (i.e., negative versus positive) of a child's socioemotional development.

Equally pivotal is the consideration of the consistent impact of prenatal maternal mental health. It is unclear whether the strength of the association between prenatal maternal mental health and developmental outcomes in offspring remains uniform across the various stages of development. As children mature, their reliance on parental influence naturally wanes, while the significance of broader sociocultural contexts beyond the confines of home gains prominence (14). In the trajectory of individual growth, peers progressively wield greater influence on socioemotional development, a phenomenon particularly conspicuous during adolescence, characterized by growing independence from parents (20). Adolescents may seek to satisfy their attachment needs through peers rather than parents (21). Consequently, it is plausible that while the influence of prenatal exposure to poor maternal mental health remains noteworthy, its strength could potentially wane. This raises the possibility that the impact of prenatal maternal mental health might vary in accordance with developmental stages, potentially receding in later periods. Alternatively, theoretical models suggest that early life adversity during specific neurodevelopmental periods steers individuals onto persistent trajectories transcending contexts (22–24). Despite its direct relevance to child development theories, this issue, to our knowledge, has yet to undergo systematic examination.

This meta-analysis holds two primary aims. Firstly, we seek to synthesize and analyze the existing body of research that explores connections between prenatal maternal mental health and developmental outcomes in offspring spanning infancy through adolescence (up to 18 years). Secondly, we endeavor to draw a comparative analysis between associations of psychological distress and positive mental health during pregnancy with offspring socioemotional development. Psychological distress includes symptoms of depression, anxiety, and stress (25). Positive mental health encompasses positive affect, satisfaction with life, optimism, and subjective well-being (26–28). Do the impacts of prenatal psychological distress differ from those of positive mental health based on the specific domain of socioemotional development? Additionally, we explore whether the associations between prenatal psychological distress or positive mental health and offspring development demonstrate consistent patterns across varying stages of development.

## Methods

### Literature search

The protocol of this meta-analysis was pre-registered in PROSPERO (ID = CRD42022335227). We searched for English language, peer-reviewed, longitudinal studies on prenatal maternal mental health and child outcomes in PsycINFO, ScienceDirect, PubMed, and EBSCOhost in June 2022 (see Supplementary Table S1 for details of the search strategy). These databases were chosen for their comprehensive coverage and accessibility to the authors. There was no restriction on date of publication. The search was limited to English language, peer-reviewed publications. The search results were compiled in Endnote and duplicates were removed before screening against the inclusion criteria. Additionally, we manually searched the reference lists of pertinent review articles to identify any relevant studies that might have been missed during the database search. Such studies were subsequently included in our analysis.

### Selection process

Articles were screened for: (a) a prospective design that spanned from pregnancy to post-pregnancy, (b) had measures of maternal mental health (e.g., depression, anxiety, positive mental health) during pregnancy, (c) had measures of the child socioemotional development (e.g., externalizing symptoms, prosocial behaviors), and (d) the child was assessed at or below age of 18 years. The inclusion and exclusion criteria followed the SPIDER checklist [(29); Supplementary Table S2].

The articles were first independently screened against the inclusion criteria by title and abstract by two reviewers. Full texts were examined if there were disagreements between the coders or it was unclear if the study was eligible to be included based on its title and abstract. The study was removed if both reviewers agreed that it did not meet the inclusion criteria. In the event of disagreement between the coders, the two coders would discuss and attempt to reach a consensus (0.02%). If disagreement persisted, the decision of the study's eligibility was determined by the first author (DYP).

### Data extraction and quality assessment

Data was extracted independently from shortlisted articles using a pre-designed data extraction form. The extracted data included: last name of first author, year of publication, country of study, name of the birth cohort (if applicable), sample size, average age of mothers during pregnancy, average gestational age, age of child during assessment, gender of child, measures of prenatal mental health, measures of child socioemotional development, and the relevant results. The extracted data was double-checked for error by a second reviewer. The developmental stage of the child was derived from the child's age based on Erikson's Psychosocial Developmental Stages. The location of the study was recoded to reflect the continent the study was situated.

It was possible for multiple effect sizes to be extracted from a study due to different developmental stage, type of prenatal mental health, and type of child outcome. If there were multiple effect sizes of the same domain of prenatal mental health, child outcome, and developmental stage within a single, an average effect size was calculated. If there were studies with overlapping samples (e.g., from the same birth cohort study), the study with more data points (e.g., provided gender-specific effects or effects across multiple developmental stages) or bigger sample size was selected. Studies that did not provide information about the measures, developmental stage of child, or independent associations between prenatal mental health and child outcomes were excluded. Studies that were included in the meta-analysis were assessed on their quality based on the NIH Quality Assessment Tool for Observational Cohort and Cross-Sectional Studies (Table 1).

**Table 1:** Characteristics of included studies.

First author (year)	Country	Sample size	Prenatal time point(s) in weeks	Age of child (years)	Developmental stage(s)	Study quality
Baibazarova et al. (30)	Netherlands	158	14–27	0.25	Infancy	11
Barker (31)	UK	12,151	32	2, 4	Toddler, preschool	12
Bekkhus et al. (32)	Norway	82,383	17, 30	3	Toddler	13
Bhat et al. (33)	India	100	32	0.21	Infancy	12
Bush et al. (34)	USA	202	12–20, 20–28	0.5	Infancy	13
Clayborne et al. (35)	Norway	36,584	17, 30	5	Preschool	10
Davids et al. (36)	USA	50	28–40	0.67	Infancy	10
Davis et al. (37)	USA	22	32	0.33	Infancy	13
Davis et al. (38)	USA	247	18–20, 24–26, 30–32	0.17	Infancy	11
Davis and Sandman (39)	USA	178	19, 25, 31	7.5	Mid childhood	12
Davis et al. (40)	USA	74	15, 19, 31, 36	12	Teen	11
de Bruijn et al. (41)	Netherlands	444	12, 24, 36	3.25	Preschool	9
Diego et al. (42)	USA	80	26	0.42	Infancy	11
DiPietro et al. (43)	USA	137	24, 28 and 32	2	Toddler	10
D'Souza et al. (44)	New Zealand	5,768	28–40	2	Toddler	12
Duguay et al. (45)	Canada	468	25	0.2	Infancy	10
Fernandes et al. (46)	India	133	28	0.17	Infancy	10
Frigerio and Nazzari (47)	Italy	90	34–36	2	Toddler	10
Galbally et al. (48)	Australia	203	28–40	4	Preschool	13
Glynn et al. (49)	USA	407	15, 19, 25, 31, 36	0.75, 2, 8.5, 13	Infancy, toddler, mid childhood, Teen	10
Gordon et al. (50)	South Africa	1,238		5	Preschool	11
Green et al. (51)	Canada	179	24–36	0.38, 1.5, 3	Infancy, toddler, preschool	11
Gustafsson et al. (52)	USA	68	14–27, 28–40	0.5	Infancy	11
Gutteling et al. (53)	Netherlands	119	15–17, 27–28, 37–38	2	Toddler	11
Guyon-Harris et al. (54)	USA	120	28–40	2	Toddler	13
Hartman et al. (55)	Norway	114,500	17, 30	5	Preschool	9
Haselbeck et al. (56)	Germany	30	12–14, 22–24, 32–34	1.33	Infancy	10
Hay et al. (57)	UK	150	14–20	13.5	Teen	13
Henrichs et al. (58)	Netherlands	2,997	20	0.5	Infancy	9
Hentges et al. (59)	Canada	1,992	<25	5	Preschool	11
Hentges et al. (60)	Canada	1,992	<25	3	Toddler	12
Huizink et al. (61)	Netherlands	170	15–17, 27–28, 37–38	0.67	Infancy	9
Huttenen et al. (62)	Finland	1,097	1–16, 17–28, 29–40	0.5, 5	Infancy, preschool	10
Korja and McMahon (63)	Australia	214	28–40	0.5	Infancy	14
Le Bas et al. (64)	Australia	1,579	<12, 12–24, 24–40	1	Infancy	12
Leerkes and Crockenberg (65)	USA	90	28–35	0.5	Infancy	10
Lin et al. (66)	USA	295	21–42	0.5	Infancy	11
Loomans et al. (67)	Netherlands	3,446	16	5	Preschool	10
Luo et al. (68)	Netherlands	687	20–25	6	Mid childhood	11
Luoma et al. (69)	Finland	147	28–40	8.5	Mid childhood	12
Martini et al. (70)	Germany	992		15.5	Teen	12
Maxwell et al. (71)	USA	1,711	14–27	10, 16	Mid childhood, teen	14
McGuinn et al. (72)	Mexico	496	18	9.5	Mid childhood	12
Mohan et al. (73)	Ireland	37	Not stated	4	Preschool	12
Nolvi et al. (74)	Finland	282	14–16, 24, 34	0.5	Infancy	10
Norcross et al. (75)	USA	259	28–40	0.5	Infancy	12
O’Connor et al. (76)	UK	7,448	18, 32	4	Preschool	13
O’Connor et al. (77)	UK	7,448	18, 32	7	Mid childhood	9
O’Donnell et al. (78)	UK	7,944	18, 32	4, 11.5	Preschool, mid childhood	13
Pacheco and Figueiredo (79)	Portugal	110	28–40	0.14	Infancy	10
Pawlby et al. (80)	UK	151	14–20, 36	16	Teen	13
Pihlakoski et al. (81)	Finland	908	10, 28	12	Teen	9
Phua et al. (19)	Singapore	1,066	26	1, 2	Infancy, toddler	9
Pickles et al. (82)	UK	813	20	3.5	Preschool	13
Polte et al. (83)	Norway	1,336	17, 32	2	Toddler	12
Porter et al. (84)	Australia	282	<20, 20–28	1	Infancy	12
Quarini et al. (85)	UK	7,959	18, 32	12	Teen	10
Rees et al. (86)	Timor-Leste	1,118	9–27	2.25	Toddler	11
Rinne et al. (87)	USA	125	20, 33	4	Preschool	11
Rodriguez and Bohlin (88)	Sweden	290	10, 12, 20, 28, 32, & 36	7	Mid childhood	11
Rothenberger et al. (89)	Germany	104	1–13, 14–27, 28–40	0.42	Infancy	9
Rouse and Goodman (90)	USA	77	9–17	0.25	Infancy	11
Rudd et al. (91)	USA	454	14–27	4	Preschool	12
Sharp et al. (92)	UK	271	20, 32	0.58	Infancy	10
Shuffrey et al. (93)	South Africa	600	20–24	3	Toddler	11
Suarez et al. (94)	Finland	407	12–40	3.7	Preschool	11
Tran et al. (95)	Vietnam	378	12–20, 28	0.5	Infancy	10
van den Heuvel et al. (96)	Infancy	90	20	0.83	Infancy	8
van den Bergh and Marcoen (97)	Belgium	71	12–22, 23–31, 32–40	8.5	Mid childhood	14
Vedova (98)	Netherlands	107	28–40	0.25	Infancy	13
Velders et al. (99)	Netherlands	2,698	20	3	Toddler	13
Wang et al. (100)	China	3,443	<12, 24–40	4	Preschool	13
Whelan et al. (101)	Australia	1,507	15	4	Preschool	12
Zhang et al. (102)	USA	153	14–40	0.5	Infancy	13

Combined developmental stages refer to pooled effect size for main analysis because the study reported effect sizes across multiple developmental stages.

### Analysis strategy

Individual effect sizes were transformed to Fisher's *Z* and analyzed in Comprehensive Meta Analysis v3 software. The results were also transformed back to Pearson *r* and reported with the *z*′ scores for ease of interpretation. The effects of negative and positive mental health on negative and positive child outcomes were analyzed separately because the directionality of effects was likely to be different. Prediction intervals for the effect sizes were also calculated if there were at least five studies in the computation of mean effect size (103). Publication bias was assessed by the funnel plot and Egger's regression test. If significant publication bias was detected, the trim-and-fill method was used to test if additional studies would change the results of the meta-analysis.

## Results

### Study sample

**Figure 1:**
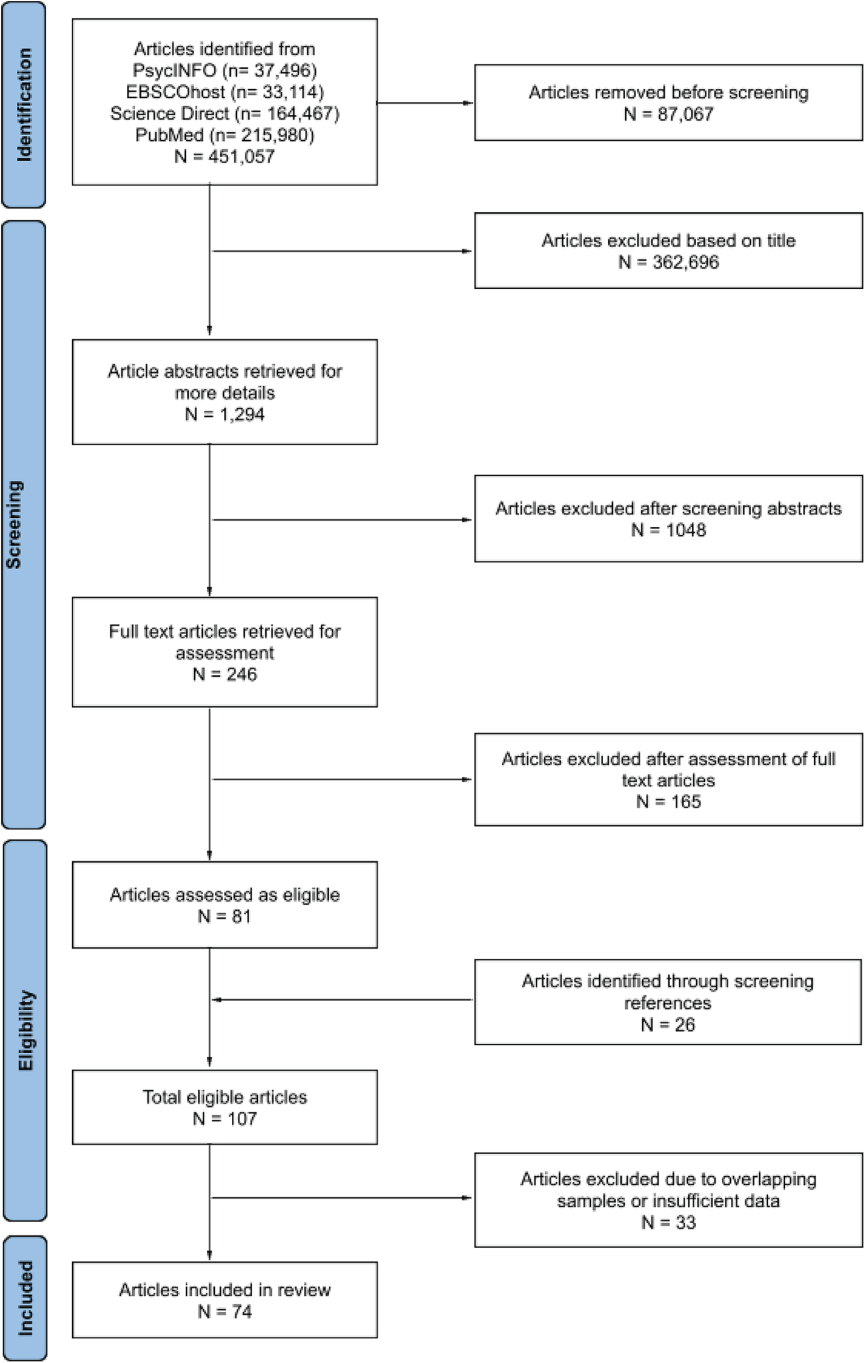
Prisma flowchart of the selection process.

There were 451,057 titles from the initial search on the four databases. Seventy-four studies with 321,966 mother-child dyads were retained for the meta-analysis (see Figure 1 for the PRISMA flowchart). The sample sizes ranged from 22 to 114,500 dyads (mean = 4,353, median = 286; see Table 1 for characteristics of included studies).

### Psychological distress on adverse outcomes

**Figure 2:**
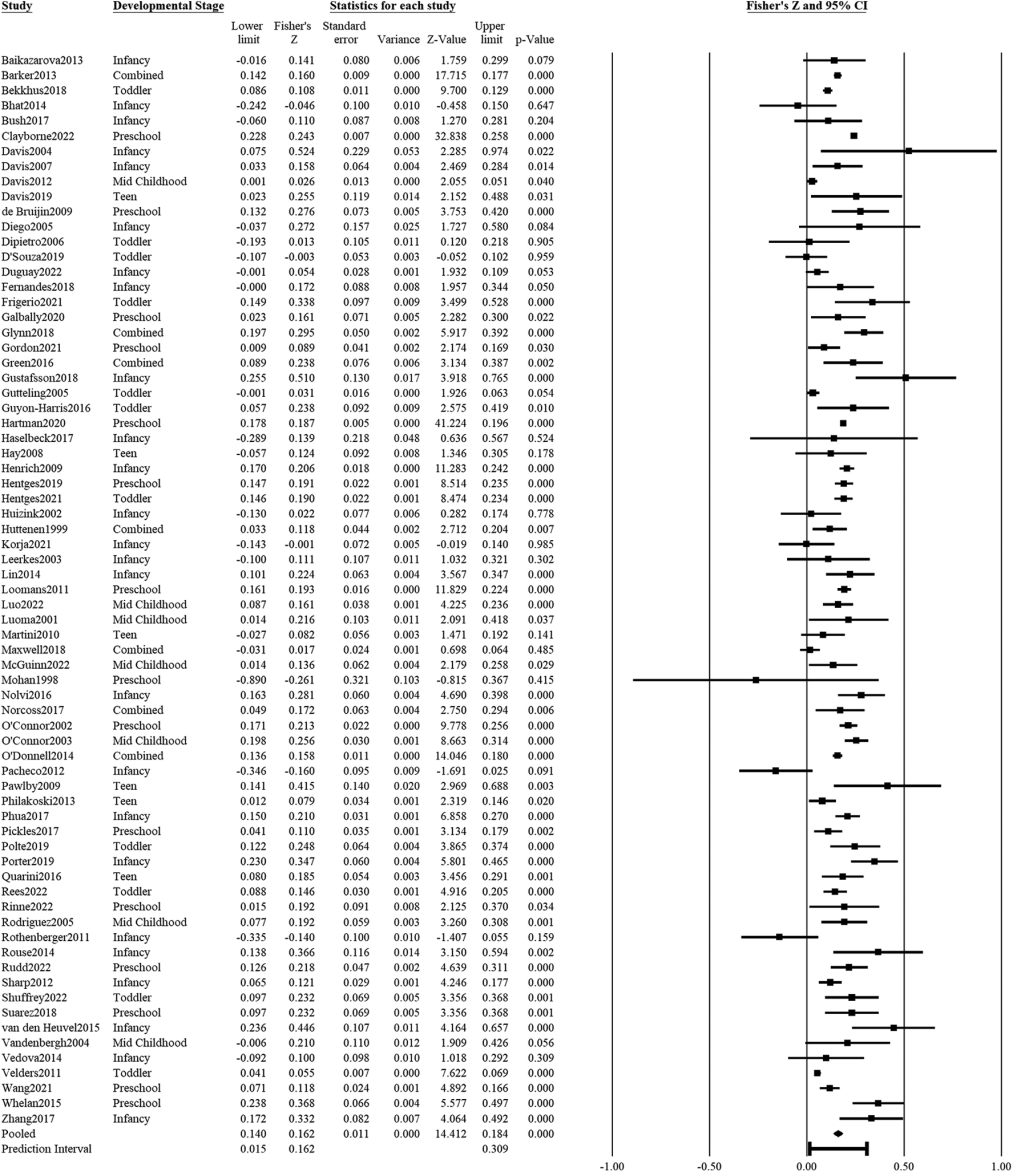
Effect sizes (in Fisher's *Z*) and forest plots for associations of prenatal maternal psychological distress and negative socioemotional outcome for the child. Combined refers to studies that had participants across multiple developmental stages and the effect sizes were averaged for this analysis.

Maternal psychological distress (e.g., depression, anxiety, stress) during pregnancy was significantly associated with adverse child outcomes (71 studies: *r *= 0.161, *z*′ = 0.162, *SE_z_*_′_ = 0.01, CI*_z_*_′_ 95% = 0.14, 0.18, *p* < .001; see Figure 2 for forest plot). The adverse child outcomes included measures of emotion dysregulation, aggressive behavior, internalizing and externalizing problems, distress to novelty, negative affect or mood, negative reactivity, depression, anxiety, fearfulness, hyperactivity, inattention, peer or behaviors problems, or socioemotional problems in general. According to the prediction interval, 95% of comparable populations would report a correlation coefficient between 0.159 and 0.299. There was significant heterogeneity in the associations of psychological distress with adverse child outcomes (*Q* = 790.59, df = 70, *p* < .001). 91.45% of the observed variance was due to variance in true effects and not due to sampling error. The funnel plot and Egger test did not detect significant publication bias in the results ($\hat{\beta }=0.018$, *t*(69) = 0.03, *p* = .97; Supplementary Figure S1A).

Ten studies provided gender-specific effect sizes of associations of prenatal psychological distress with adverse child outcomes. There was no significant difference in the effect sizes of the associations of psychological distress with adverse outcomes between male (*r *= 0.196, *z*′ = 0.1988, *SE_z_*_′_ = 0.020, CI*_z_*_′_ 95% = 0.160, 0.238) and female (*r* = 0.196, *z*′ = 0.199, *SE_z_*_′_ = 0.022, CI*_z_*_′_ 95% = 0.155, 0.243) children (*Q* < 0.001, df = 1, *p* = .99). Due to the low number of studies that provided gender-specific data, we were not able to estimate if the gender differences in effect sizes differ across different developmental stages.

Two meta-regression models were estimated to examine the moderating effect of developmental stages and location on the effect sizes of associations of prenatal psychological distress with adverse child outcomes. With infants as the reference group, none of the developmental stages had a significant moderating effect on the size of the associations of psychological distress with either adverse or positive child outcomes (−0.03 < *β*s < 0.03, *p*s* *> .30). Developmental stage accounted for 13% of the total between-study variance. With North America as the reference group, the continent where the data was collected did not significantly moderate the effect of psychological distress on adverse child outcomes (−0.04 < *β*s < −0.002, *p*s* *> .40). Location accounted for almost 0% of the between-study variance of effect sizes.

### Psychological distress on positive outcomes

**Figure 3:**
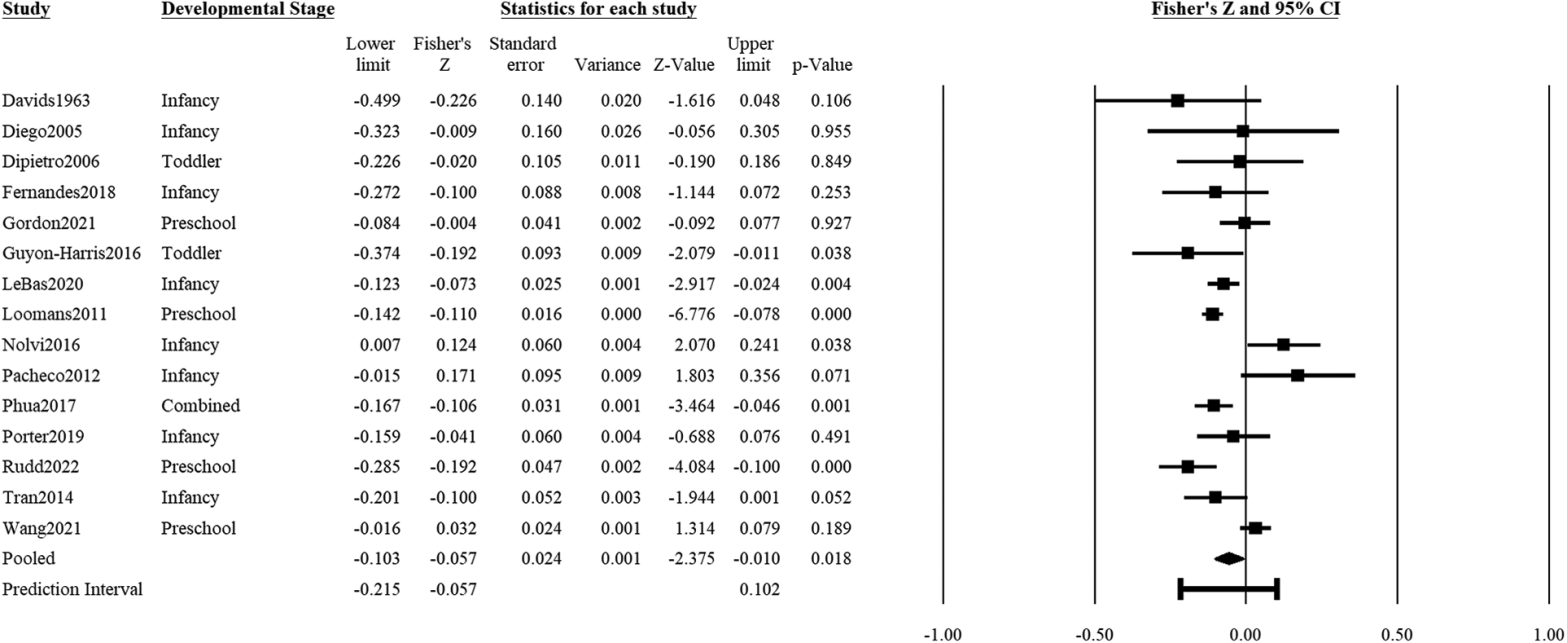
Effect sizes (in Fisher's *Z*) and forest plots for associations of prenatal maternal psychological distress and positive socioemotional outcome for the child. Combined refers to studies that had participants across multiple developmental stages and the effect sizes were averaged for this analysis.

Maternal psychological distress during pregnancy showed a significant and inverse association with positive child outcomes (15 studies: *r *= *z*′ = −0.06, *SE_z_*_′_ = 0.02, CI 95% = −0.10, −0.01, *p* = .018; Figure 3). Positive child outcomes included adaptive skills, prosocial behaviors or relations, positive affect or mood, empathy, excitability, positive reactivity, or general socioemotional competence. The prediction interval for 95% of comparable populations was between *r* = −0.09 and 0.05. There was significant heterogeneity in effect sizes of the associations of psychological distress with positive child outcomes (*Q* = 55.61, df = 14, *p* < .001), with 74.8% of the variance in effect sizes being true variance. The funnel plot and Egger test did not detect significant publication bias ($\hat{\beta }=0.53$, *t*(13) = 0.57, *p* = .58; Supplementary Figure S1B).

Two studies provided gender-specific effects of psychological distress on positive child outcomes. As such, a subgroup analysis by gender was not possible. In addition, no studies reported the effect of psychological distress on outcomes in mid-childhood and adolescence. With infancy as the reference group, there was no significant difference in effect size between outcomes of infants vis-à-vis outcomes of toddlers (*β* = −0.09, CI 95% = −0.27, 0.10, *p* = .37) nor preschoolers (*β* = −0.04, CI 95% = −0.14, 0.07, *p* = .50). With North America as the reference group, studies in Europe reported significantly bigger effect size than studies in North America (*β* = 0.16, CI 95% = 0.03, 0.29, *p* = .022); the effect sizes of studies in other continents were not significantly different from North American studies (*p*s > 0.09).

### Positive mental health on adverse outcomes

**Figure 4:**
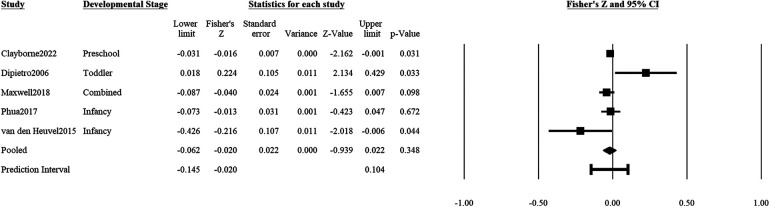
Effect sizes (in Fisher's *Z*) and forest plots for associations of prenatal maternal positive mental health and negative socioemotional outcome for the child. Combined refers to studies that had participants across multiple developmental stages and the effect sizes were averaged for this analysis.

Positive maternal mental health (e.g., uplifts or positive mood) during pregnancy was not significantly associated with adverse child outcomes (5 studies: *r* = *z*′ = −0.02, *SE_z_*_′_ = 0.02, CI*_z_*_′_ 95% = −0.06, 0.02, *p* = .35; Figure 4). The prediction interval of effect sizes was estimated to be between *r* = −0.19 and 0.083. There was significant heterogeneity in associations of positive prenatal mental health with adverse child outcomes (*Q* = 9.66, df = 4, *p* = .047), with true variance in effect sizes estimated to be 58.6% of what was collated in this study. There was no indication of publication bias based on the funnel plot and Egger test ($\hat{\beta }=-0.05$, *t*(3) = 0.04, *p* = .97; Supplementary Figure S1C).

Only one study (35) reported a gender-specific effect size of positive prenatal mental health on adverse child outcomes. There were also insufficient studies to conduct meta-regressions on the moderating effect of developmental stage or continent.

### Positive mental health on positive outcomes

**Figure 5:**
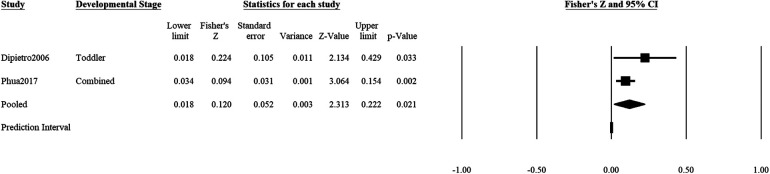
Effect sizes (in Fisher's *Z*) and forest plots for associations of prenatal maternal positive mental health and positive socioemotional outcome for the child. Combined refers to studies that had participants across multiple developmental stages and the effect sizes were averaged for this analysis.

Positive maternal mental health during pregnancy was significantly associated with positive child outcomes (2 studies: *r* = *z*′ = 0.12, *SE_z_*_′_ = 0.05, CI 95% = 0.003, 0.018, *p *= .021; Figure 5). An accurate estimation of the prediction interval was not possible due to the low number of studies (104). *Q*-statistic showed that there was no significant heterogeneity in the effects of positive prenatal mental health and positive child outcomes (*Q *= 1.41, df = 1, *p* = .24), but 71.1% of the observed variance was likely to be due to sampling error (*I*^2^ = 28.94). The non-significant heterogeneity is likely due to the low number of studies.

## Discussion

This meta-analysis aimed to comprehensively explore the nuanced associations between maternal psychological distress and positive mental health during pregnancy and the diverse facets of child socioemotional development, encompassing various developmental stages, domains of development, and geographical locations. Importantly, our investigation revealed no significant evidence of publication bias or moderation by study location, underscoring the robustness of our findings.

Our study has significantly contributed to the discourse by discerning that the impact of maternal psychological distress and positive mental health during pregnancy exhibits specificity with respect to child outcomes. Specifically, our results indicate that prenatal psychological distress exhibits stronger associations with adverse child outcomes, whereas positive prenatal mental health demonstrates stronger associations with positive outcomes. This distinct pattern highlights the independence of negative and positive mental health constructs (9, 10, 19), substantiating the need for separate investigations into maternal mental ill-health and well-being. Furthermore, these findings underscore the necessity of exploring both negative and positive developmental trajectories in children to provide a more comprehensive understanding of their socioemotional development.

Intriguingly, while prenatal psychological distress exhibited significant associations with child outcomes, the association was notably weak. This aligns with prior meta-analyses that similarly reported modest associations between poor prenatal mental health and various child outcomes. Maternal prenatal anxiety was significantly, but weakly, associated with preterm births and low birthweight (105). In another meta-analysis, the odds of adverse socioemotional development for children with mothers who experienced prenatal depression and anxiety were likewise small, with odds ratios of 1.76 and 1.47, respectively (106). Beyond the prenatal period, maternal depression also displayed weak correlations with socioemotional problems (0.15 < *r*s < 0.24) and positive affect (*r *= −0.10) in children (107). Thus, our study underscores the multi-faceted nature of influences on child socioemotional development, emphasizing the importance of considering a broad range of factors in future research.

Comparably, the association between positive prenatal mental health and positive child outcomes rivaled the effect size observed for the association between psychological distress and adverse outcomes. Particularly, the effect size for positive prenatal mental health was even greater than that for the association between psychological distress and positive outcomes. This finding echoes existing literature demonstrating the distinct impacts of positive and negative maternal mental health on specific child outcomes. For instance, positive antenatal maternal mental health is associated with positive cognitive, language, social, and competence development of the child, with no corresponding significant association for negative antenatal mental health (19). This absence of overlap in predictors of socioemotional difficulties and well-being has also been observed in adolescent samples (17). Thus, our study accentuates the necessity of embracing a comprehensive understanding of maternal mental health across the entire spectrum and its influence on child development by incorporating measurements of positive development.

A pivotal finding from our analysis emerged in the context of developmental stages. Contrary to our initial expectations, the strength of the association between psychological distress and adverse socioemotional development remained consistent across developmental stages. This consistency is somewhat surprising given our anticipation that the effects of prenatal mental health might diminish over time due to mitigating factors from the broader sociocultural environment. It is plausible that prenatal mental health may set individuals or families on trajectories that perpetuate adverse early life circumstances (22–24). The persistence of prenatal ill-health symptoms beyond pregnancy into childhood supports this notion (108–110).

Due to the limited number of studies, our analyses predominantly centered on the relationship between prenatal psychological distress and adverse child outcomes. This emphasis on the negative spectrum of mental health and development mirrors the current state of the field. While health is intrinsically characterized by complete well-being and not just the absence of illness, perinatal mental health or well-being is often operationalized solely as the nonexistence of depressive or anxiety symptoms (111). We echo the call from fellow researchers in advocating for an expansion of our exploration into maternal mental health and child development to encompass the positive spectrum of constructs (11, 13, 15, 17, 112).

### Limitations

This meta-analysis must be interpreted in light of the limitations. Foremost, the scarcity of studies delving into positive mental health and child outcomes constrained our ability to conduct exhaustive analyses on these dimensions. Second, the diverse mental health and socioemotional development measures were amalgamated into overarching negative and positive categories. For example, there was no differentiation between depression and anxiety (i.e., psychological distress), optimism and positive affect (i.e., positive mental health), internalizing and externalizing symptoms (i.e., negative socioemotional outcome), nor prosocial behaviors and positive affectivity (i.e., positive socioemotional outcome). This oversimplification might not accurately capture the nuances that certain aspects of mental health or child development hold. These complexities could potentially be moderated by factors such as gender, developmental stages, and dimensions that were not encapsulated in this meta-analysis. Longitudinal research has revealed gender-specific differences in the risk and protective factors for internalizing and externalizing symptoms during adolescence (113). The intricate nuances outlined above signify a significant research gap, impinging on the depth of our comprehension of the intricate relationship between maternal mental health and child development.

Furthermore, our study's scope was confined to the pregnancy period, preventing us from investigating the endurance of prenatal mental health symptoms and their subsequent impact on child development. However, extant data from various birth cohorts suggest a substantial persistence of maternal depression and anxiety symptoms from pregnancy into childhood (114–116). Notably, children whose mothers experienced enduring mental health symptoms throughout the peripartum period displayed elevated odds of manifesting internalizing problems in comparison to their peers (117).

It is also essential to recognize that child development is inherently multifactorial and complex. While this study emphasizes the significance of prenatal mental health as a potential influential factor, child development is shaped by a myriad of interrelated influences, including genetics, environmental factors, and broader familial dynamics. Ultimately, a child's socioemotional well-being develops from an intricate interplay of genetic, environmental, and maternal factors. Our study contributes to the expanding body of evidence that highlights the significance of maternal mental health during pregnancy, particularly emphasizing the distinctive contribution of positive mental health to child socioemotional development.

## Conclusion

This study validates the existing body of literature by reaffirming the substantial link between prenatal maternal mental health and the socioemotional development of children. Moreover, the observed associations between prenatal psychological distress and child outcomes, while significant, were consistently weaker than anticipated, urging us to unveil the moderating influences at play. An illustrative instance of such moderation is evident in the work of Goodman et al. (107), which showed the impact of maternal depression symptoms on offspring socioemotional development being amplified within families facing limited economic resources.

By unveiling the distinct impacts of psychological distress and positive mental health across domains and developmental stages, our findings advocate for a comprehensive approach to maternal well-being and child development. It is imperative that we broaden our perspective to encompass the positive spectrum of both mental health and child development. While averting adverse socioemotional outcomes remains pivotal, nurturing the positive socioemotional growth of children assumes equal significance. The contexts for interventions and public health initiatives aimed at preventing ill-health and promoting well-being diverge significantly, necessitating distinct strategies to maximize the potential of the next generation.

## Data Availability

The raw data supporting the conclusions of this article will be made available upon request to the corresponding author, without undue reservation.
