# Supplementary material for: Differential effects of prenatal psychological distress and positive mental health on offspring socioemotional development from infancy to adolescence: a meta-analysis
Source: Front Pediatr. 2023 Sep 14;11:1221232. doi: 10.3389/fped.2023.1221232 (PMC10536167; doi:10.3389/fped.2023.1221232)

# Supplementary Tables

## Table S1. Details of the search strategy.

Keywords:

(Prenatal OR pregnan* OR maternal OR trimester OR perinatal OR mother), AND

(child OR offspring OR adol*), AND

(depress* OR dysthymn* OR affective OR negative mood OR anxiety OR anxious OR mood disorder OR internalizing OR stress OR distress OR negative emotion* OR mental health OR externalizing OR aggress* OR opposition* OR conduct problems OR hostil* OR socioemotional development OR social competence OR social withdrawal OR loneliness OR optimis* OR positive mental health OR gratitude OR resilience OR happ* OR life satisfaction OR positive affect OR well-being)

NOT rat

NOT intervention

NOT qualitative

NOT trial

NOT review

## Table S2. Inclusion and exclusion criterion according to SPIDER guidelines.

| **SPIDER** | Inclusion Criteria | Exclusion Criteria |
| --- | --- | --- |
| **Population** | Mother-offspring dyad  Offspring ≤ 18 years old | Mother data only  Father data  Offspring data only  Children older than 18 years old  Chronic medical condition (e.g., cancer)  Animal studies |
| **Phenomenon of Interest** | Prenatal maternal mental health  Offspring internalizing, externalizing, social and positive mental health development | Postnatal maternal data  Not socioemotional data for child  Not mental health data for mother |
| **Design** | Longitudinal design | - Cross-sectional - Intervention - Quasi-experiment - Randomized controlled trials |
| **Evaluation** | Mother self-report of mental health  Mother report of child development  Child self-report of development | Father reported data  Non-mother reported data on maternal mental health |
| **Research type** | Quantitative  Articles with abstract and full text in  English | Qualitative  Case studies  Conference proceedings  Non-peer-reviewed (e.g., thesis or dissertation)  Reviews or meta-analysis |

# Supplementary Figures

## Figure S1. Funnel plots of the analyses

(A) Prenatal maternal psychological distress with negative child outcomes


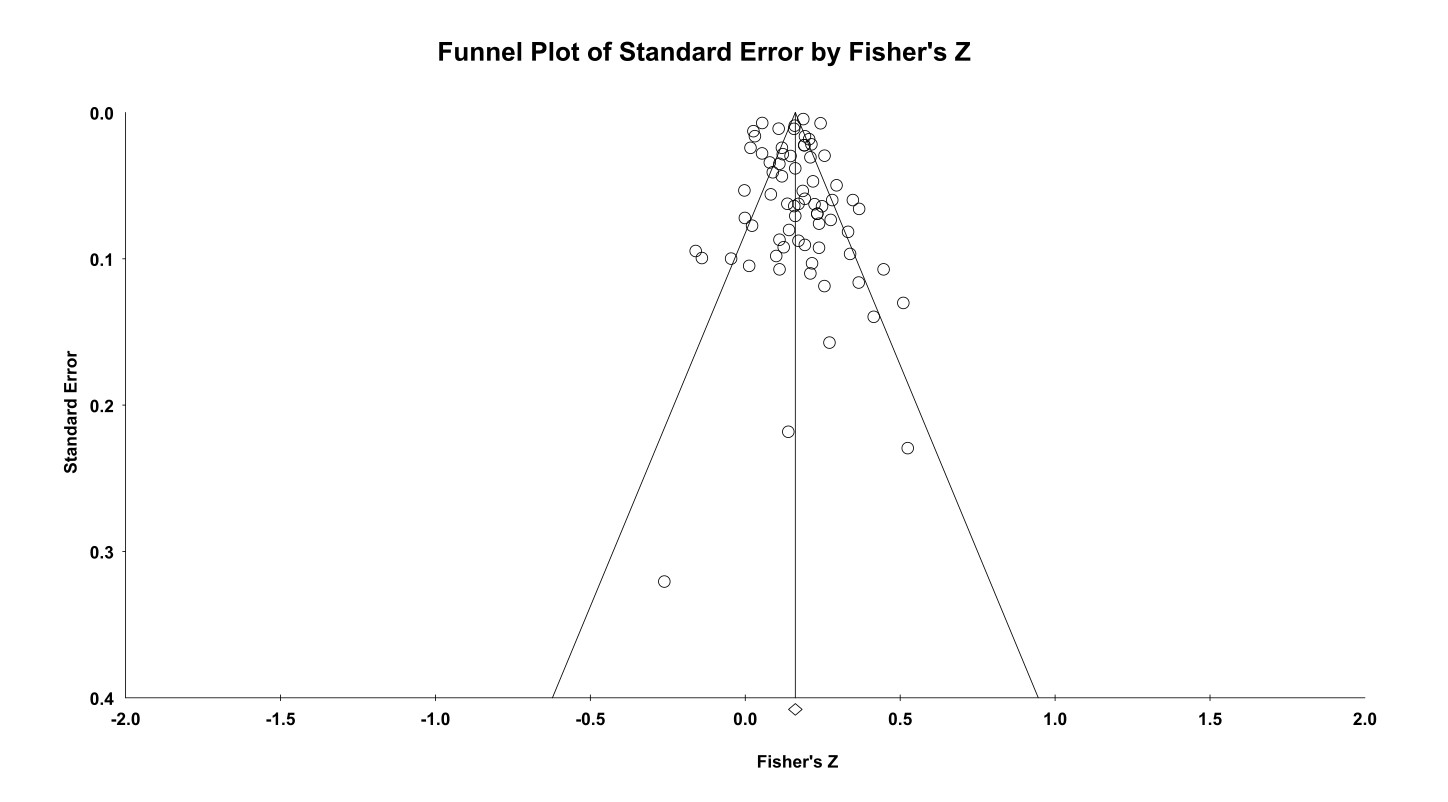


(B) Prenatal maternal psychological distress with positive child outcomes


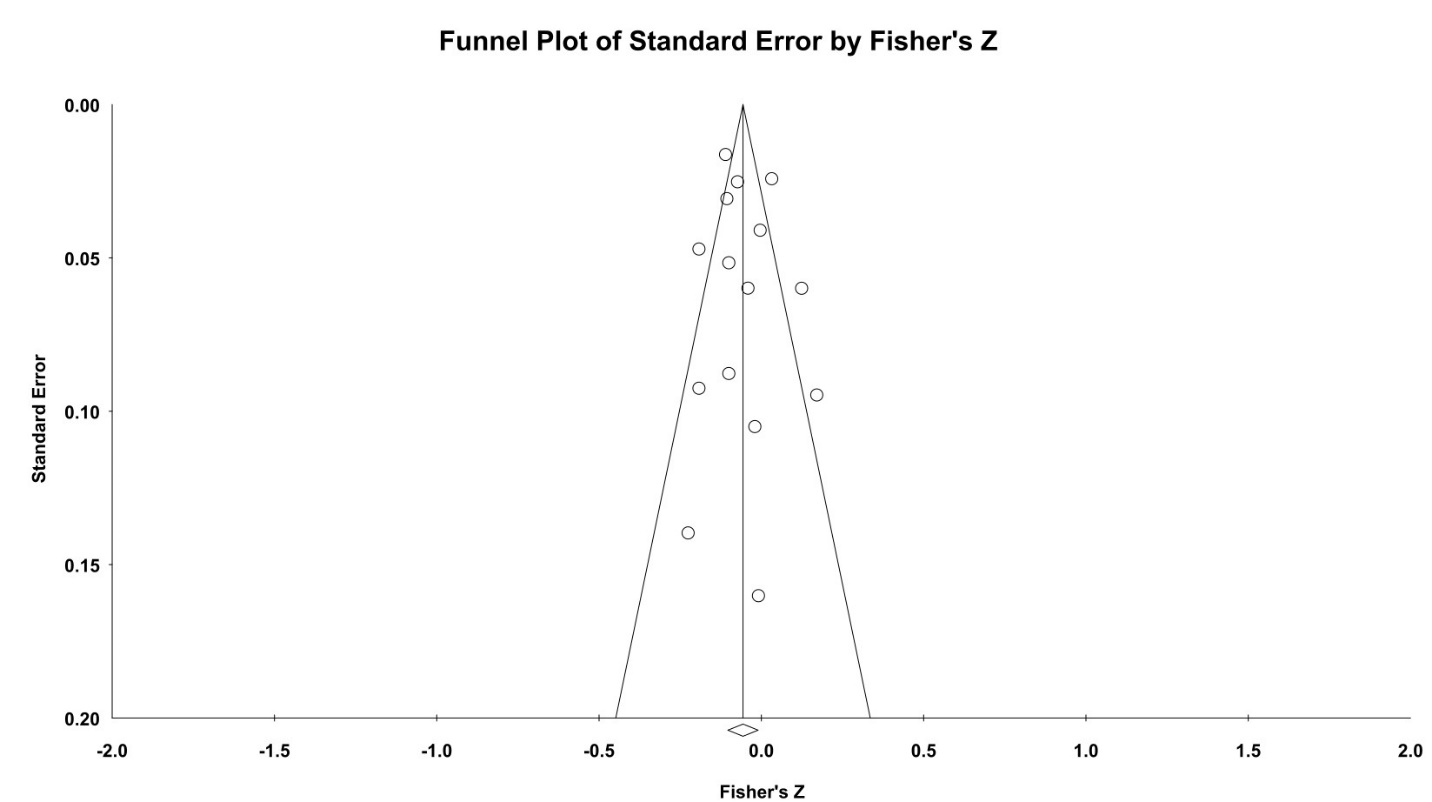


(C) Positive prenatal maternal mental health with negative child outcomes


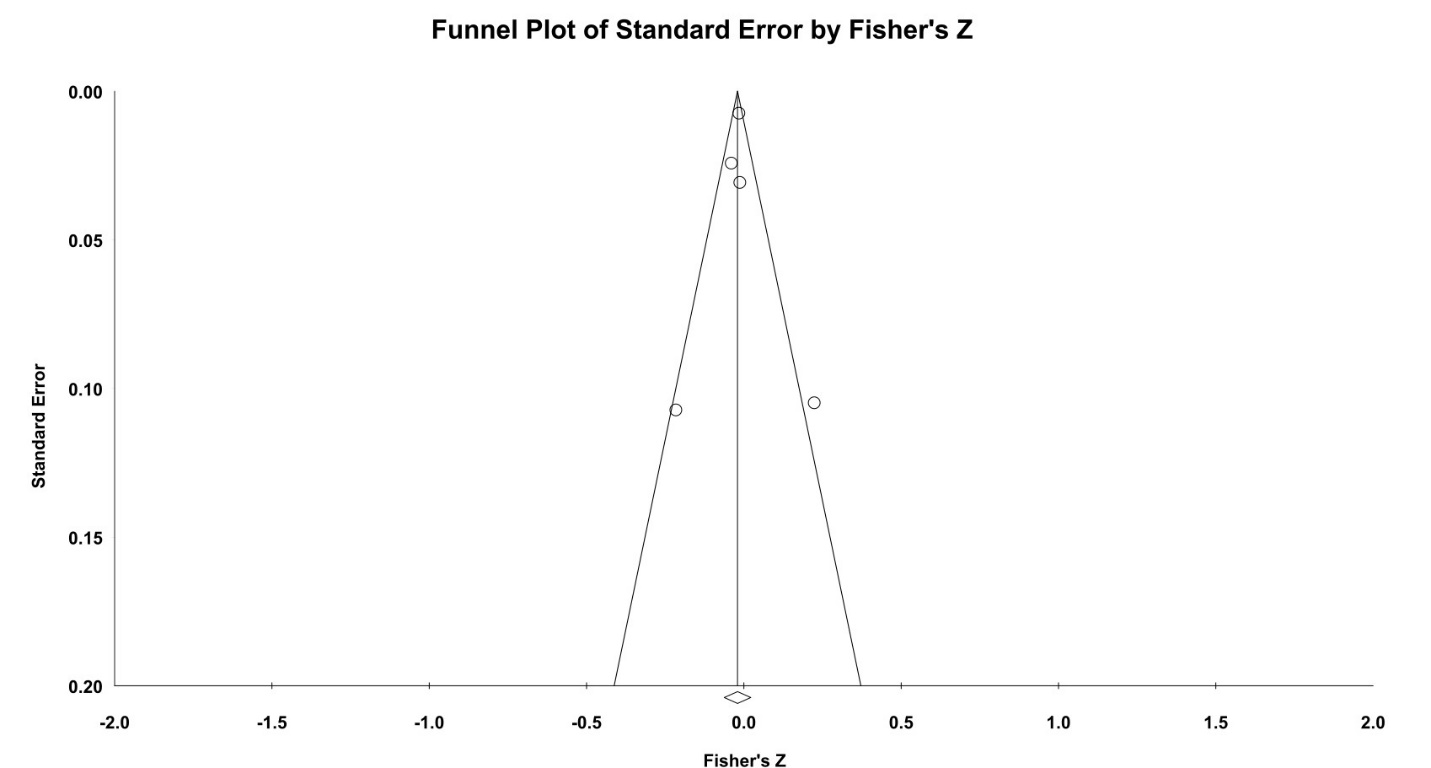

Supplement: Supplementary file 1 [file Datasheet1.docx]
